# Perceptions of Vaccination in Latin America: Insights from Panama, the Dominican Republic, and Guatemala

**DOI:** 10.7759/cureus.103779

**Published:** 2026-02-17

**Authors:** Asis A Babun, Cassidy Carda, Kelson Knighton, Johannes du Randt, Mariana Keech, Maxwell Monson, Isabel Sirrine, Thomas Bigham, Mark L Wardle

**Affiliations:** 1 Military Medicine, Rocky Vista University College of Osteopathic Medicine, Ivins, USA; 2 Global Health, Rocky Vista University College of Osteopathic Medicine, Ivins, USA; 3 Biomedical Sciences, Rocky Vista University College of Osteopathic Medicine, Ivins, USA; 4 Primary Care Medicine, Rocky Vista University College of Osteopathic Medicine, Ivins, USA

**Keywords:** belief over vaccines, confidence on vaccine uptake, covid-19 vaccine perception, global public health, vaccine compliance, vaccine safety

## Abstract

Public perceptions of vaccines were evaluated through a survey conducted by medical students from Rocky Vista University in March 2025. The survey included 252 patients attending medical outreach clinics in Panama, the Dominican Republic, and Guatemala. Results revealed a surprisingly high self-reported vaccination rate of 77.8% across the three countries, reflecting widespread vaccine acceptance and uptake. More than half of participants (54.4%) identified clinics or hospitals as their primary source of vaccine-related information, positioning health institutions at the forefront of information dissemination. Although 57.9% of respondents rated access to vaccines as easy overall, suggesting that physical access may not represent a major barrier, the survey highlighted other key obstacles, including fears of side effects and insufficient information. These findings provide valuable guidance for targeted health communication initiatives and may inform the development of broader strategies to address community-level barriers to vaccination and improve overall uptake.

## Introduction

Vaccine hesitancy remains a major barrier to public health initiatives worldwide and significantly affects vaccination coverage across diverse populations, including communities in Latin America and the Caribbean [1,2]. Multiple studies have shown that vaccine hesitancy in this region is driven by a complex interplay of factors, including mistrust of healthcare systems, concerns about vaccine safety, misinformation, and sociocultural beliefs [2,3]. These challenges became particularly evident during the COVID-19 pandemic, as varying levels of trust in public institutions and healthcare systems influenced vaccine acceptance in countries such as the Dominican Republic, Panama, and Guatemala [1,4].

Understanding community-specific perceptions and barriers is essential to developing effective and sustainable vaccination strategies. Prior research indicates that tailored, culturally sensitive interventions are more effective than generalized approaches in addressing vaccine hesitancy and improving immunization uptake [5-7]. In Latin America, structural barriers, including limited access to healthcare services, disparities in vaccine distribution, and gaps in public health communication, further exacerbate hesitancy and contribute to suboptimal vaccination rates [2,8-10].

The present study evaluates perceptions of vaccination and examines the barriers that individuals in communities in Panama, Guatemala, and the Dominican Republic face when making vaccine-related decisions. The primary objective was to conduct a descriptive, cross-sectional assessment of self-reported vaccination attitudes, perceived barriers, and sources of vaccine information among adults attending medical outreach clinics. By identifying behavioral, cultural, and contextual factors influencing vaccine hesitancy in these populations, this study aims to inform the development of targeted communication and intervention strategies [4-6]. Given the convenience sampling approach and recruitment within healthcare outreach clinics, the findings are intended to characterize this clinic-attending population and should not be interpreted as nationally representative. Rather, the results are exploratory and hypothesis-generating, intended to guide future population-based research and public health planning efforts [8,9].

## Materials and methods

Study design, location, duration, and population

A comprehensive, cross-sectional study was conducted during a Global Medical Outreach in Panama, Guatemala, and the Dominican Republic. Using an anonymous, survey-based approach, the study assessed adult participants’ perceptions of vaccination, barriers to vaccination, and informational needs at outreach clinics.

Data were collected contemporaneously during clinic operations in March 2025, representing diverse communities across Latin America and the Caribbean. Participants included adults attending the medical outreach clinics in these locations.

Inclusion and exclusion criteria

Participants were eligible if they were adults aged 18 years or older, could understand the survey in Spanish (or with the assistance of a translator), provided informed consent, and attended one of the Global Medical Outreach clinic sites during the study period.

Exclusion criteria included age under 18, inability to provide informed consent, cognitive or severe mental impairments preventing comprehension of the survey, or unwillingness or inability to complete the survey.

Sampling technique

A convenience sampling method was used, with participants recruited in person at medical outreach clinics and invited to complete a paper-based questionnaire voluntarily. No incentives were offered, and refusal to participate had no negative consequences.

Survey instrument and data collection

The study authors developed the questionnaire specifically for this study and did not adapt it from any previously published or proprietary instrument. Originally drafted in English, the survey was translated into Spanish and verified by a native Spanish speaker to ensure suitability for the study population.

Nine Spanish-translated questionnaire forms were incorporated into an anonymous survey protocol. The survey was designed to assess vaccine access, trust in healthcare systems, primary sources of vaccine information, degree of vaccine hesitancy, and specific support needs. Questions focused exclusively on individual perceptions and beliefs regarding vaccination, with no invasive or emotionally distressing items included.

For participants in Guatemala who did not speak Spanish, a trained Mayan interpreter assisted with survey administration to ensure comprehension.

Ethical considerations

The study protocol, survey instruments, and all supporting documents were reviewed and approved by the Rocky Vista University Institutional Review Board on February 10, 2025 (RVU IRB #2024-256). Participation was entirely voluntary, with no monetary or material compensation provided. Surveys were completed anonymously, and no personally identifiable information was collected, ensuring participant confidentiality and privacy. Patient health and well-being were prioritized throughout the data collection process.

Statistical analysis

Survey responses were analyzed using descriptive statistical methods. Categorical variables were summarized as frequencies and percentages. All analyses were performed using Python version 3.14.2 (Python Software Foundation, Wilmington, DE, USA) to characterize trends in vaccine perceptions, access, trust, and hesitancy across the surveyed populations.

## Results

**Figure 1:**
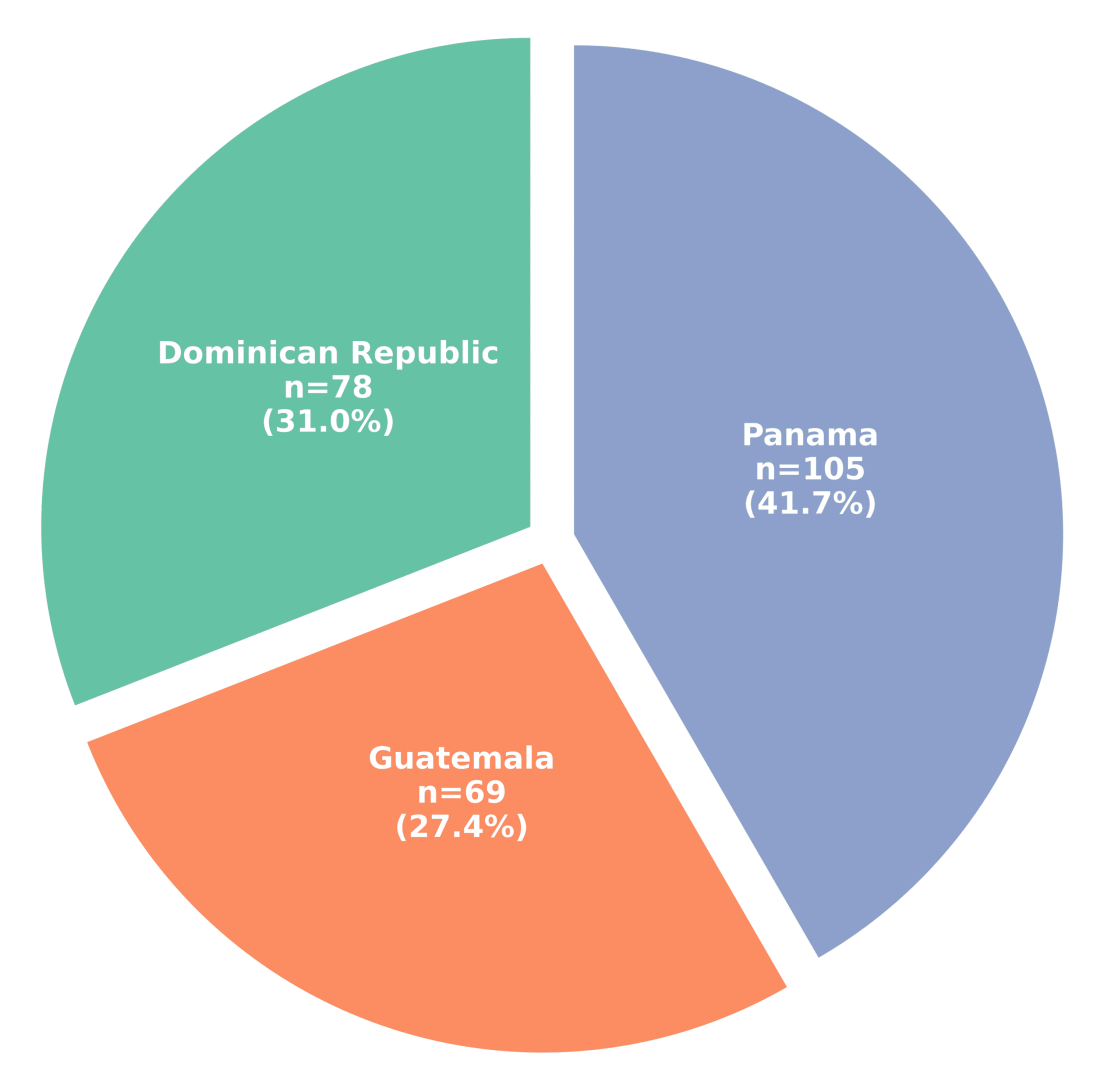
Distribution of survey respondents across three countries in Latin America

**Figure 2:**
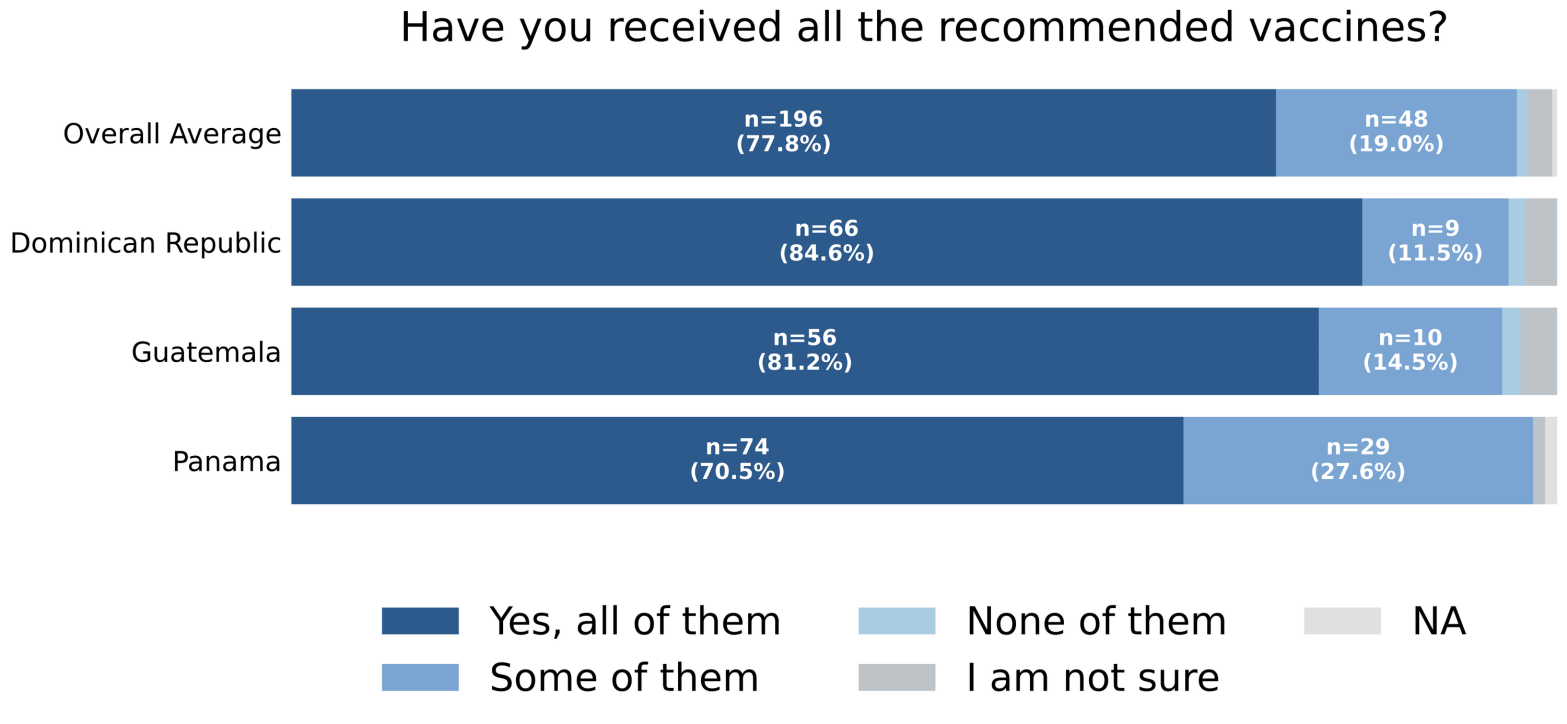
Vaccination compliance Self-reported response to “Have you received all the recommended vaccines?” by country and overall. One participant did not respond to this question and was classified as "NA". NA: not applicable

A total of 252 participants completed the survey: 105 (41.7%) from Panama, 78 (31.0%) from the Dominican Republic, and 69 (27.4%) from Guatemala (Figure 1). Regarding self-reported vaccination history, 196 (77.8%) reported receiving all recommended vaccines, 48 (19.0%) reported receiving some, two (0.8%) reported receiving none, and five (2.0%) were unsure of their vaccination status (Figure 2).

**Figure 3:**
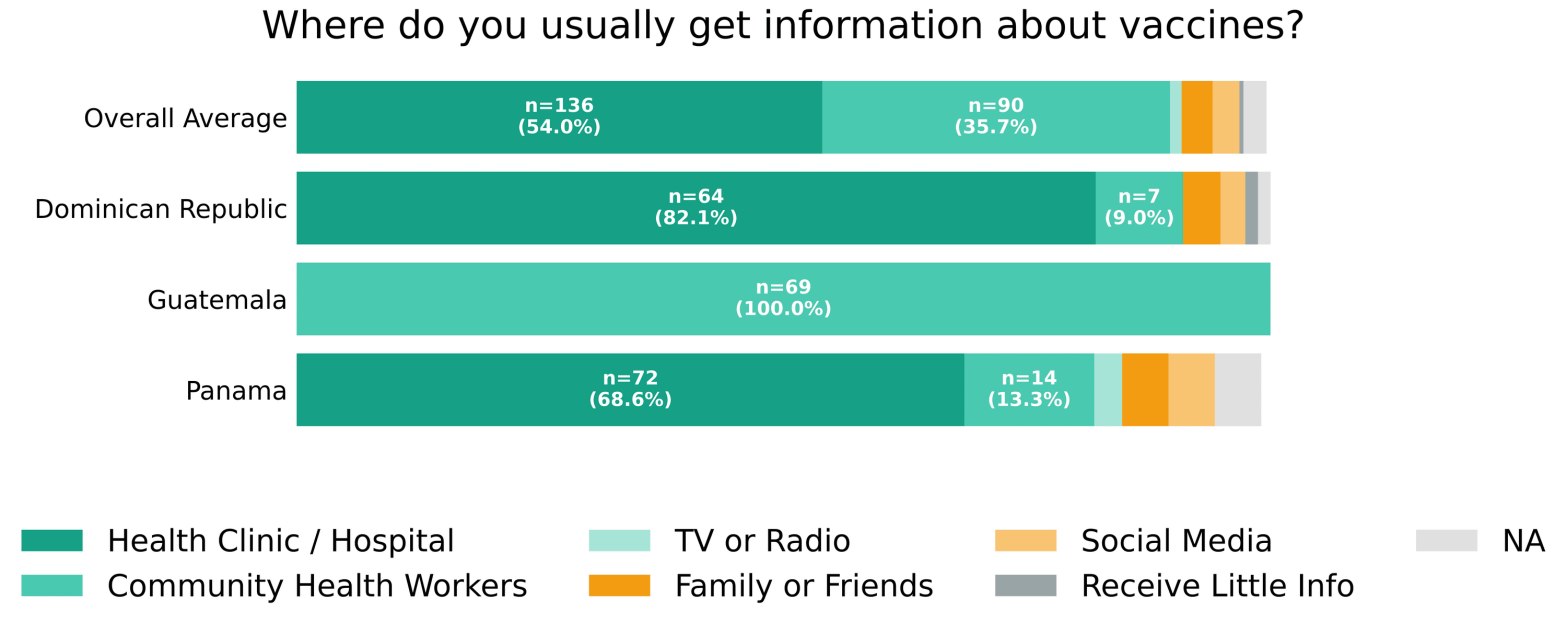
Vaccine information sources Self-reported response to “Where do you usually get information about vaccines?” by country and overall. Respondents selecting multiple answer choices (n = 5) or who did not respond to the question (n = 1) are classified as "NA". NA: not applicable

**Figure 4:**
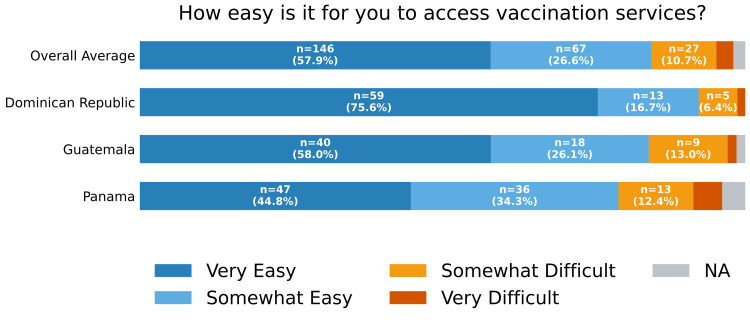
Access to vaccine services Self-reported response to “How easy is it for you to access vaccination services?” by country and overall. Respondents selecting multiple answer choices (n = 1) or who did not respond to the question (n = 4) are classified as "NA". NA: not applicable

The most common sources of vaccine information were health clinics or hospitals (136; 54.0%) and local community healthcare workers (90; 35.7%) (Figure 3). Access to vaccination services was perceived as "very easy" by 146 (57.9%) and "somewhat easy" by 67 (26.6%). A smaller proportion reported access as "somewhat difficult" (27; 10.7%) or "very difficult" (7; 2.8%) (Figure 4).

**Figure 5:**
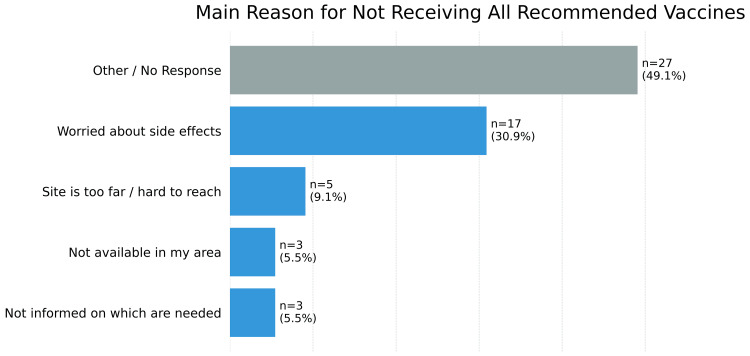
Reason for not receiving all of the recommended vaccines Respondents who reported not receiving all the recommended vaccines (n = 55) were asked, “What is the main reason for not getting a vaccine (for yourself or your child) if you haven't?"

Among all participants, the most frequently cited reason for not receiving a vaccine was concern about side effects (17; 30.9%). Other reasons included not being informed about required vaccines (3; 5.5%) and the vaccination site being too far or difficult to reach (5; 9.1%). A substantial proportion either specified "other" reasons or did not respond (27; 49.1%) (Figure 5).

**Figure 6:**
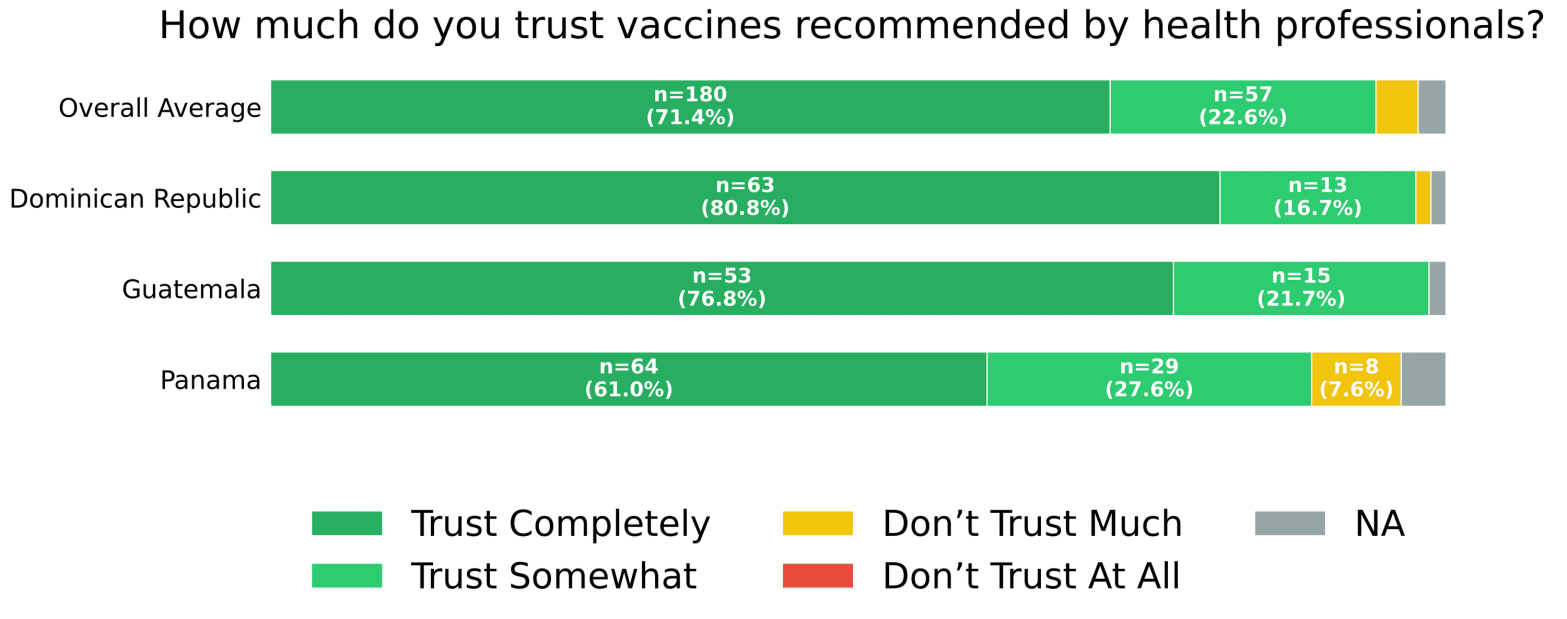
Trust of recommended vaccines Self-reported responses to "How much do you trust vaccines recommended by health professionals?" overall and by country. Six respondents did not answer the question and were classified as "NA". NA: not applicable

**Figure 7:**
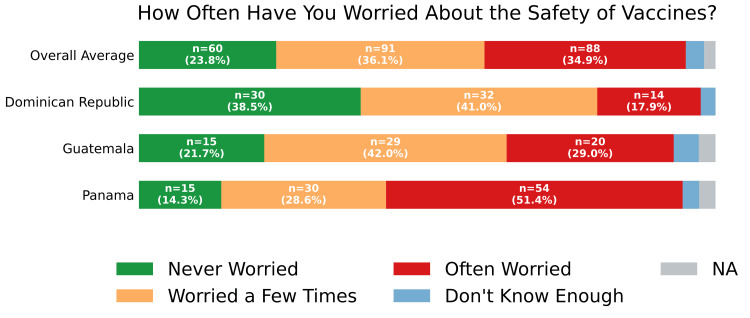
Safety perception of vaccines Self-reported response to "Have you ever been worried about the safety of vaccines?" overall and by country. Four respondents did not answer the question and were classified as "NA". NA: not applicable

**Figure 8:**
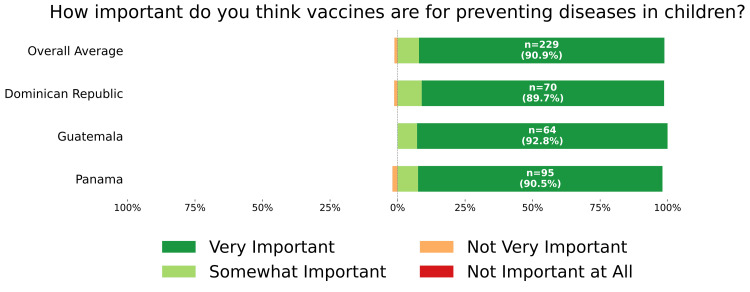
Importance of vaccines in preventing diseases in children Self-reported response to "How important do you think vaccines are for preventing diseases in children?" NA: not applicable

Overall trust in vaccines recommended by health professionals was high, with 180 participants (71.4%) reporting that they "trust them completely" and 57 (22.6%) reporting that they "trust them somewhat" (Figure 6). Responses regarding vaccine safety were more varied: 91 participants (36.1%) reported worrying only occasionally, 88 (34.9%) reported worrying often, and 60 (23.8%) reported never having worried (Figure 7). Despite these concerns, an overwhelming majority (229; 90.9%) considered vaccines for preventing diseases in children to be "very important" (Figure 8).

## Discussion

Most survey results suggest that attitudes toward vaccination are largely positive in the Dominican Republic, Guatemala, and Panama. Respondents in these countries also demonstrated a relatively high degree of trust in healthcare providers. Previous studies have indicated that skepticism toward health recommendations from unofficial sources may increase vaccine hesitancy, whereas trust in the recommending institution is a major contributing factor [1]. The survey results from these countries suggest that trust is a crucial public health strategy for improving vaccination awareness. Building such trust enables public health efforts to address barriers to vaccine uptake stemming from misinformation and cultural misunderstandings, which remain common despite widespread vaccine access. Given that healthcare workers are trusted sources of information, enhanced education campaigns and outreach programs can address both healthcare providers’ concerns about vaccine side effects and information gaps [2].

Nonetheless, vaccine safety concerns and limited information remain key barriers to vaccine uptake in the Dominican Republic, Guatemala, and Panama. Fear of side effects, often associated with false beliefs about vaccine components such as thimerosal and aluminum adjuvants, continues to hinder vaccination [3]. This fear is particularly prevalent in certain population cohorts, including women and non-binary individuals [4]. Misinformation about vaccine components is fueled by limited safety information and general mistrust, particularly among economically disadvantaged rural populations [4]. Targeted interventions should include educational campaigns that inform communities about vaccine safety and efficacy, and counter misinformation about vaccine components through trusted healthcare providers.

Calibrated strategies are necessary to increase vaccine access in the Dominican Republic, Guatemala, and Panama. First, establishing mobile clinics can improve access by reducing transportation barriers, particularly in remote or marginalized areas [5]. Healthcare worker outreach programs can be expanded to build community trust and directly address misconceptions about vaccine safety and necessity. Educators can conduct campaigns to inform the public about vaccinations and provide reliable information, leveraging the credibility of health professionals [6]. These interventions are targeted rather than permanent solutions, but their implementation is likely to significantly benefit vulnerable populations in Panama, Guatemala, and the Dominican Republic.

Moreover, the study’s findings on perceptions of vaccination in these countries have important public health implications for the future health of Latin America. When planning vaccine communication and mobilization strategies, regional and cultural nuances should be carefully considered. Social elements, such as local credible sources and social networks, can substantially influence vaccine decision-making [7]. Future communication and mobilization interventions should therefore account for regional cultural characteristics to ensure that health promotion messages resonate with potential vaccine recipients. Lessons from HPV vaccination programs show that providing vaccines simultaneously at health centers and schools can help overcome logistical barriers, such as the observed “drop-off” in completion rates [8]. Understanding and addressing the regional cultural context can strengthen future communication and programming strategies, thereby improving vaccination coverage and preventing public health crises in Latin America.

It is also important to consider the limitations of this study in the context of vaccine perceptions. The study was conducted in healthcare settings, which naturally include populations that actively seek medical care and trust healthcare providers. Another limitation is the relatively small sample size (n = 252), which restricts analysis across population subgroups in the Dominican Republic, Guatemala, and Panama. Additionally, self-reported responses may be affected by biases, such as recall bias and social desirability bias, which could influence reported vaccine coverage and perceived barriers [9]. Future studies should consider larger sample sizes and longitudinal designs to better understand vaccine perceptions over time. Incorporating determinants of vaccine coverage identified in prior research could further enhance understanding and guide interventions targeting these communities [10].

In the context of vaccination in the Dominican Republic, Guatemala, and Panama, the scenario highlights interrelated challenges affecting public health, particularly in relation to migration through areas such as the Darien Gap. Migrants may experience disruptions in access to healthcare due to their transient status, which may exacerbate vaccine hesitancy and hinder full vaccination coverage. Vaccine strategies should therefore be tailored to migrants’ lifestyles, migration status, mobile health units, and cross-border partnerships [11]. This can be achieved by identifying partners and developing approaches that leverage existing international collaborations while adopting culturally specific communication methods for each country. Such approaches should be inclusive of migrants and sensitive to the host country’s socioeconomic context, ensuring that migration does not compromise vaccination efforts and that infectious disease outbreaks are prevented both at destination sites and in other regions.

## Conclusions

The proposed targeted strategies to increase vaccination coverage in the Dominican Republic, Guatemala, and Panama should focus on addressing vaccine safety concerns and information gaps. Cultural perceptions of vaccination in these countries are largely positive, as reflected by high trust in healthcare providers. Nevertheless, fears about vaccines and limited access to reliable information continue to hinder full vaccination uptake. High-priority interventions, such as healthcare worker outreach programs and mobile clinics, should be implemented to overcome both access and informational barriers. These initiatives can help build community trust through direct, in-person communication and support incremental improvements in vaccination coverage. Although these findings are exploratory and hypothesis-generating, they may inform future public health initiatives to reduce cultural and economic barriers to vaccination and improve health outcomes in Panama, the Dominican Republic, and Guatemala. Larger population-based studies are warranted before these insights can inform regional policy decisions.

## Appendices

Questionnaire: English

1. Have you received all the recommended vaccines?

a) Yes, all of them

b) Some of them

c) None of them

d) I am not sure

2. Where do you usually get information about vaccines?

a) Health clinic or hospital

b) Local community health workers

c) Family or friends

d) Social media

e) Television or radio

f) I don’t receive much information about vaccines

3. How easy is it for you to access vaccination services?

a) Very easy

b) Somewhat easy

c) Somewhat difficult

d) Very difficult

4. What is the main reason for not getting a vaccine (for yourself or your child) if you haven't?

a) I don’t think vaccines are necessary

b) Vaccination site is too far or hard to reach

c) I am worried about side effects

d) Vaccines are not available in my area

e) I am not informed about which vaccines are needed

f) Other (please specify)

5. How important do you think vaccines are for preventing diseases in children?

a) Very important

b) Somewhat important

c) Not very important

d) Not important at all

6. How much do you trust vaccines recommended by health professionals?

a) I trust them completely

b) I trust them somewhat

c) I don’t trust them much

d) I don’t trust them at all

7. What would encourage you to get you or your child vaccinated?

a) More information about the benefits of vaccines (benefits)

b) A recommendation by your health care provider (recommendation/trust)

c) More information on vaccine safety (safety)

d) Easier access to vaccines (access)

e) Nothing, I am already convinced

8. Have you ever been worried about the safety of vaccines?

a) Yes, often

b) Yes, but only a few times

c) No, I have never worried

d) I don’t know enough to be concerned

9. What kind of support in your community do you feel is needed to help you and your children get vaccinated (more than one answer is possible)?

a) More information from health professionals

b) Mobile vaccination clinics

c) Reducing the cost of transportation

d) Health workers visiting homes

e) I don’t need additional support

Questionnaire: Spanish

1. ¿Has recibido todas las vacunas recomendadas?

a) Sí, todas

b) Algunas

c) Ninguna

d) No estoy seguro/a

2. ¿Dónde sueles obtener información sobre las vacunas?

a) Clínica o hospital

b) Trabajadores de salud de la comunidad

c) Familia o amigos

d) Redes sociales

e) Televisión o radio

f) No recibo mucha información sobre las vacunas

3. ¿Qué tan fácil es para ti acceder a los servicios de vacunación?

a) Muy fácil

b) Algo fácil

c) Algo difícil

d) Muy difícil

4. ¿Cuál es la principal razón por la que no te has vacunado (a ti o a tu hijo/a)?

a) No creo que las vacunas sean necesarias

b) El lugar de vacunación está muy lejos o es difícil de alcanzar

c) Me preocupa los efectos secundarios

d) Las vacunas no están disponibles en mi zona

e) No sé qué vacunas son necesarias

f) Otra (especifica)

5. ¿Qué tan importante crees que son las vacunas para prevenir enfermedades en los niños?

a) Muy importante

b) Algo importante

c) No muy importante

d) Nada importante

6. ¿Cuánto confías en las vacunas recomendadas por los profesionales de salud?

a) Confío completamente

b) Confío algo

c) No confío mucho

d) No confío nada

7. ¿Qué te animaría a ti o a tu hijo/a a vacunarse?

a) Más información sobre los beneficios de las vacunas

b) Una recomendación de tu Doctor

c) Más información sobre la seguridad de las vacunas

d) Acceso más fácil a las vacunas

e) Nada, ya estoy convencido/a

8. ¿Alguna vez te has preocupado por la seguridad de las vacunas?

a) Sí, bastante

b) Sí, pero solo algunas veces

c) No, nunca me he preocupado

d) No sé lo suficiente para preocuparme

9. ¿Qué tipo de ayuda en tu comunidad crees que se necesita para ayudar a que tú y tus hijos se vacunen? (puedes escoger más de una respuesta)

a) Más información de los doctores

b) Clínicas móviles de vacunación

c) Reducir el costo del transporte

d) Trabajadores de salud visitando casas

e) No necesito más ayuda
